# Kefir-Derived Postbiotics Inhibit the Periodontal Pathogen *Campylobacter rectus* through Combined Organic Acid and Proteinaceous Antimicrobial Mechanisms

**DOI:** 10.4014/jmb.2607.07002

**Published:** 2026-09-21

**Authors:** Young-Seon Kim, Hyunsook Kim, Wallace H. Yokoyama, Nitin Nitin, Kun-Ho Seo

**Affiliations:** 1 Center for One Health, College of Veterinary Medicine, Konkuk University, Seoul 05029, Republic of Korea; 2 Department of Food and Nutrition, Hanyang University, Seoul 04763, Republic of Korea; 3 USDA Agricultural Research Service, Western Regional Research Center, Albany, CA 94710, USA; 4 Department of Food Science and Technology, University of California, Davis, CA 95616, USA

**Keywords:** Postbiotics, Kefir-derived lactic acid bacteria, *Campylobacter rectus*, Organic acids, Bacteriocin-like protein, Periodontal pathogen

## Abstract

Postbiotic metabolites from lactic acid bacteria (LAB) offer antimicrobial functionality without the safety and stability constraints of live probiotics. Here, we evaluated cell-free supernatants from kefir-derived LAB against the periodontal pathogen *Campylobacter rectus* and characterized their inhibitory components. Four kefir isolates (*Lactobacillus kefiranofaciens* DD2 and DN1, *Lentilactobacillus kefiri* DH5, and *Leuconostoc mesenteroides* DH1608) suppressed *C. rectus* growth, with maximal inhibition after 72 h culture at 30°C. The antimicrobial activity was largely retained under acidic conditions, but reduced after heat treatment in DD2 and DH5, indicating heat-labile inhibitory component(s) in addition to organic acids. Enzymatic treatment revealed a proteinaceous component in DD2 metabolites, as proteases (proteinase K and trypsin) significantly reduced inhibitory activity. High-performance liquid chromatography (HPLC) quantification confirmed high lactic acid levels (285.77–359.83 mM) and elevated propionic acid (26–28 mM) in all supernatants. Lactic acid (300 mM) and propionic acid (25 mM) individually inhibited *C. rectus* growth. A protein-/polypeptide-enriched fraction from *L. kefiranofaciens* DD2 (highest yield: 12.58 ± 0.77 mg/L) showed antimicrobial activity (48.16 ± 13.27%), and tricine–SDS-PAGE revealed an approximately 28-kDa band within this fraction. However, the active component(s) were not molecularly identified, and further activity-guided purification and LC–MS/MS analysis are required. Collectively, kefir-derived LAB postbiotics inhibit *C. rectus* growth through combined organic acid and proteinaceous mechanisms, supporting their development as postbiotic candidates for oral health applications targeting this periodontal pathogen.

## Introduction

Chronic periodontal disease is characterized by bacteria-induced inflammation of the gum tissues surrounding the teeth, leading to gingival inflammation, destruction of the periodontal ligament, and loss of alveolar bone [1, 2]. Periodontal disease is prevalent in companion animals, affecting a substantial proportion of the pet population [3, 4]. Early plaque biofilm in a healthy oral cavity is dominated by gram-positive and facultative aerobic coliforms and cocci. However, the onset of gingivitis is associated with an increased prevalence of gram-negative and anaerobic species [5]. Despite decades of studies on canine periodontal bacteria [6-10], the microbial species that cause periodontal disease remain unclear. Certain species, including *Actinomyces* sp., *Porphyromonas cangingivalis*, *Campylobacter* sp., and *Porphyromonas gulae* have been frequently isolated from dogs with periodontitis, with *P. gulae* being notably abundant [11]. Additionally, *P. gulae*, *Tannerella forsythia*, and *Campylobacter rectus* have been identified in the oral cavities of pets and their owners [12].

Scaling, a common preventive treatment for periodontal disease, only offers temporary relief from plaque and tartar. Additionally, it carries significant risk owing to the need for anesthesia, making it a potentially hazardous procedure for companion animals [13, 14]. Other preventive methods, such as tooth brushing, demand constant effort from pet owners [15]. The use of triclosan, an antimicrobial agent found in toothpaste, may inadvertently increase bacterial resistance, contributing to the emergence of antibiotic-resistant strains [16]. These challenges highlight the increasing need for alternative preventive measures against periodontal disease.

Recently, postbiotics—beneficial metabolites and non-viable bacterial components produced by probiotics—have gained recognition as functional alternatives that circumvent the safety, stability, and regulatory challenges inherent in administering live cultures [17, 18]. Postbiotics exert diverse biological effects, including immunomodulation, antibacterial, antioxidant, and anticancer activities [19]. Lactic acid bacteria (LAB)-derived postbiotics represent a particularly promising class of antimicrobial metabolites [20]. Antimicrobial agents such as organic acids, polysaccharides, and antimicrobial peptides (bacteriocins) inhibit the growth of pathogenic bacteria [21, 22]. Organic acids, including acetic, citric, and lactic acids, exhibit antibacterial activity against gram-negative bacteria, and their efficacy is contingent on their composition and concentration [23-25]. Polysaccharides, known for their high biosafety, exert antioxidant and antimicrobial effects in a dose-dependent manner, although their mechanisms of action remain poorly understood [26, 27].

In our previous studies, we demonstrated that kefir exhibits broad antimicrobial activity against various foodborne pathogens and spoilage bacteria [28]. We also isolated kefir-derived LAB, including *Lentilactobacillus kefiri* DH5 and *Lactobacillus kefiranofaciens* DN1 and DD2, and characterized their antimicrobial activities using culture supernatants and/or bioactive fractions such as exopolysaccharides [29, 30]. In addition, inhibitory and anti-biofilm activities of *Leuconostoc mesenteroides* and *L. kefiranofaciens* DN1/DD2 against oral pathogens have been reported [31, 32]. However, the mechanisms by which kefir-derived LAB postbiotic metabolites inhibit *C. rectus* have not been characterized. Therefore, in this study, we sought to delineate how combined organic acids and proteinaceous antimicrobial activity contribute to growth inhibition of *C. rectus*, positioning kefir-derived postbiotics as functional alternatives to live probiotics in oral health contexts.

## Materials and Methods

### Bacterial Strains and Preparation of Postbiotics

Four kefir-derived LAB strains were used: *L. kefiranofaciens* DD2 and DN1, *L. kefiri* DH5, and *L. mesenteroides* DH1608. These strains were previously isolated from kefir-fermented milk and maintained as stock cultures in our laboratory collection based on their documented functional properties [29-31].

Each stock strain was streaked onto de Man–Rogosa–Sharpe (MRS) agar and incubated at 37°C for 48–72 h. A single colony was inoculated into MRS broth and cultured to the stationary phase. The cultures were then standardized to an initial inoculum of 1 × 10^6^ CFU/mL and incubated under three conditions: 30°C for 48 h, 30°C for 72 h, or 37°C for 72 h. At the end of incubation, the bacterial cells were removed by centrifugation at 4,000 × *g* for 15 min at 4°C, and the supernatants were collected and sterilized by filtration through a 0.22-μm membrane filter (Merck Millipore, USA) to obtain cell-free postbiotic preparations. Based on the initial antimicrobial screening, the cell-free supernatants produced at 30°C for 72 h were used for subsequent characterization experiments. The filtrates were stored at -20°C until use.

### Target Pathogen and Antimicrobial Assays

*Campylobacter rectus* KCOM 3972, a periodontal pathogen, was obtained from the Korean Collection for Oral Microbiology (KCOM; Republic of Korea). The strain was cultured in nutrient broth at 37°C for 48 h under anaerobic conditions.

The antimicrobial activity of the LAB cell-free supernatants and derived fractions was assessed using a *C. rectus* growth-curve assay. For the growth-curve assay, 100 μL of the LAB supernatant was mixed with 800 μL of nutrient broth, and the mixture was inoculated with 100 μL of *C. rectus* prepared in nutrient broth to yield an initial density of ≥1 × 10^5^ CFU/mL. A 200 μL aliquot of each preparation was dispensed into a 96-well plate, and optical density at 600 nm (OD_600_) was measured at 1 h intervals using a Multiskan FC microplate reader (Thermo Fisher Scientific, USA) at 37°C for 24 h. Clindamycin (100 μg/mL) was used as a positive control and MRS broth as a negative control. Antimicrobial efficacy (%) was calculated from OD values relative to the controls.

### Heat, pH, and Enzyme Sensitivity Tests

To characterize their physicochemical stability and infer the biochemical nature of the inhibitory components, LAB cell-free supernatants produced at 30°C for 72 h were subjected to heat, pH, and enzyme treatments. For the heat stability test, aliquots were heated at 100°C for 10 or 30 min or autoclaved at 121°C for 15 min. For the pH stability test, the pH of each supernatant was measured using a pH meter (Orion Star A211 pH Desktop Meter, Thermo Fisher Scientific) and adjusted to 2–9 with 1 M HCl or 1 M NaOH.

For enzyme sensitivity tests, the supernatants were mixed with α-amylase (in 50 mM phosphate buffer), trypsin (in 50 mM Tris–HCl buffer), pepsin (in 20 mM acetate buffer), lipase, or proteinase K to a final enzyme concentration of 2 mg/mL. The mixtures were incubated at 37°C for 4 h, followed by boiling for 10 min to inactivate the enzymes.

Residual antimicrobial activity of the LAB cell-free supernatants against *C. rectus* was assessed using the growth-curve assay described in Section Target Pathogen and Antimicrobial Assays. Antimicrobial efficacy (%) was calculated by comparing OD_600_ values at 24 h with those of the relevant control condition, and untreated supernatants processed in parallel served as untreated controls.

### HPLC Analysis of Organic Acids

To quantify the antimicrobial organic acids in the LAB cell-free supernatants, the organic acid profiles of the LAB cell-free supernatants were determined by high-performance liquid chromatography (HPLC). Organic acid standards (acetic, citric, lactic, and propionic acids) were obtained from Sigma-Aldrich (USA) and used for peak identification and quantification. Analyses were performed on an Agilent 1260 HPLC system equipped with ChemStation software (Agilent, USA) using an Aminex HPX-87H column (300 × 7.8 mm, 9 μm; Bio-Rad Laboratories, USA).

The mobile phase was 5 mM sulfuric acid (Daejung Chemicals & Metals, Republic of Korea) delivered for 40 min at a flow rate of 0.6 mL/min. The injection volume was 10 μL, and the column temperature was maintained at 30°C. Eluted organic acids were detected at 214 nm. Prior to analysis, standards and the mobile phase were filtered through 0.45 μm syringe filters and 0.20 μm membrane filters (Advantec, Japan), respectively.

Based on the HPLC results, lactic and propionic acids that were present at higher levels in the LAB metabolites than those in uninoculated MRS broth were selected for antimicrobial validation. Lactic acid (300 mM) and propionic acid (25 mM) were prepared in MRS broth and evaluated for anti-*C. rectus* activity using the microplate assay described in Section Target Pathogen and Antimicrobial Assays. For these organic acid validation assays, inhibition of *C. rectus* growth was determined using an endpoint criterion (i.e., absence of detectable growth at 24 h compared with the corresponding control condition), rather than by reporting full growth-curve parameters. Comparisons were made against the matched uninoculated MRS control; a separate pH-matched (non-organic acid) control was not included.

### Preparation and Characterization of PP Fractions

Purified PP fractions were prepared from the LAB cell-free supernatants by ammonium sulfate precipitation. Briefly, the supernatants were brought to 70% saturation with ammonium sulfate under overnight stirring, followed by centrifugation (3,924 × *g*, 20 min, 4°C). The resulting pellet was dissolved in 0.05 M phosphate buffer, filtered through a 0.45 μm syringe filter, and dialyzed (1 kDa cutoff; Spectrum Spectra/Por Biotech-Grade RC Dialysis Membrane Tubing, Thermo Fisher Scientific) against distilled water for 24 h. The dialyzed fraction was lyophilized using an FD freeze dryer to obtain PP powder.

PP concentration was determined using the Bradford protein assay with bovine serum albumin as a standard, and the PP yield was expressed as mg/L of the original MRS culture. For antimicrobial testing and electrophoretic analysis, the PP powder was reconstituted in 0.05 M phosphate buffer and filtered through a 0.45 μm syringe filter.

The anti-*C. rectus* activity of the purified PP fractions was evaluated using a 96-well microplate growth-inhibition assay adapted from established microplate bacteriocin screening protocols [33]. Briefly, lyophilized PP fractions were reconstituted in 0.05 M phosphate buffer to the highest soluble concentration obtainable for each preparation (therefore, final test concentrations differed among strains) and sterilized by 0.45 μm syringe filtration. For each assay, 10 μL of the filtered PP solution was added to 800 μL of pre-reduced nutrient broth, and the mixture was inoculated with 100 μL of an actively growing *C. rectus* suspension prepared in nutrient broth to obtain an initial density of ≥ 1 × 10^5^ CFU/mL. Aliquots of this mixture (200 μL) were dispensed into sterile 96-well plates, which were then incubated anaerobically at 37°C with OD_600_ measured at 1 h intervals for 24 h using a Multiskan FC microplate reader (Thermo Fisher Scientific). Phosphate buffer (no PP) served as the negative control, and clindamycin (100 μg/mL) served as the positive control. Antimicrobial efficacy (%) of the PP fractions was calculated from the OD_600_ values at 24 h relative to the buffer control (Section Target Pathogen and Antimicrobial Assays).

The molecular weight profile of the most active PP fraction was assessed by tricine–sodium dodecyl sulfate-polyacrylamide gel electrophoresis (SDS-PAGE) using 16% separating and 4% stacking gels in a vertical electrophoresis system (Bio-Rad Laboratories). Purified PP and molecular weight markers were run at 100 mA. Gels were stained with Coomassie Brilliant Blue R-250 (Bio-Rad Laboratories) and destained with 40% (v/v) methanol and 10% (v/v) acetic acid, and the apparent molecular weight of the PP fraction was estimated relative to the marker.

### Statistical Analysis

All experiments were performed in triplicate. Data are presented as mean ± standard deviation. Differences among groups were analyzed using one-way analysis of variance (ANOVA) with Tukey’s post hoc test, and *p*-values < 0.05 were considered statistically significant.

## Results

### Antimicrobial Activity of the Kefir-Derived LAB Supernatants against *C. rectus*

Fig. 1 shows the growth kinetics of *C. rectus* in nutrient broth supplemented with the LAB cell-free supernatants from four kefir-derived LAB isolates, *L. kefiranofaciens* DD2 and DN1, *L. kefiri* DH5, and *L. mesenteroides* DH1608, produced under different incubation conditions: 48 h at 30°C, 72 h at 30°C, and 72 h at 37°C. The negative control (MRS broth) supported robust growth of *C. rectus*, reaching a maximal OD_600_ of approximately 0.7–0.8, whereas clindamycin (positive control) maintained OD_600_ near baseline throughout the assay.

Across all isolates, the LAB cell-free supernatants produced at 30°C for 72 h consistently yielded the strongest inhibition. Notably, supernatants from *L. kefiranofaciens* DD2 and DN1 prepared under this condition resulted in complete suppression of detectable growth over 24 h (panels A and B). In contrast, supernatants produced at 30°C for 48 h or 37°C for 72 h showed partial inhibition, with reduced final OD_600_ values compared with the negative control (approximately 0.2–0.5 depending on strain and condition). For *L. kefiri* DH5 and *L. mesenteroides* DH1608, the 30°C/72 h supernatants produced the lowest growth levels (final OD_600_ ~0.3–0.5), and the DH1608 supernatant under the most inhibitory condition also delayed the onset of growth, extending the lag phase to approximately 10–12 h (panel D). Collectively, these results indicate that supernatant production at 30°C for 72 h maximizes anti-*C. rectus* activity, with *L. kefiranofaciens* DD2 and DN1 exhibiting the most pronounced inhibition among the tested kefir isolates.

### Stability of the Antimicrobial Activity of LAB Metabolites under Heat and pH

The physicochemical stability of the kefir-derived LAB metabolites was evaluated by assessing their residual inhibition of *C. rectus* growth after heat and pH treatment. The native pH of the kefir-derived LAB postbiotics was acidic, ranging from pH 3.67–4.47 (Fig. 2): *L. kefiranofaciens* DN1 (pH 3.67) and DD2 (pH 3.94) were more acidic, whereas *L. kefiri* DH5 (pH 4.47) and *L. mesenteroides* DH1608 (pH 4.39) had pH values above 4, consistent with organic acid production during fermentation. Under acidic conditions (pH 2–4), anti-*C. rectus* activity was largely maintained (typically ≥90% efficacy), supporting a major contribution of acid-mediated inhibition. Upon neutralization and alkalinization (pH 8–9), the inhibitory activity of the LAB metabolites decreased in a strain-dependent manner; notably, DH5 and DH1608 retained partial activity under basic conditions (approximately 40–60% efficacy), suggesting the presence of additional non-acid inhibitory component(s) in these strains (Fig. 3).

Heat stability also differed by strain (Fig. 4). Supernatants of DD2 showed a significant reduction in antimicrobial efficacy after heating at 100°C (10–30 min) and after autoclaving at 121°C for 15 min (*p* < 0.05), with efficacy falling below approximately 80% relative to the untreated supernatant. Similarly, DH5 exhibited a significant loss of activity after heat treatment (*p* < 0.05). In contrast, the inhibitory activity of DN1 was largely unchanged after heating at 100°C and remained high (>90%). Similarly, DH1608 also retained measurable activity after heating at 100°C (residual efficacy >60%). Collectively, these patterns indicate that acid-mediated inhibition is broadly important across strains, whereas DD2 and DH5 likely produce an additional heat-labile inhibitory factor(s) beyond organic acids.

### Enzyme Sensitivity Suggests a Proteinaceous Component in DD2 Metabolites

To further infer the biochemical nature of the inhibitory LAB metabolites, the kefir-derived LAB supernatants were treated with hydrolytic enzymes and residual anti-*C. rectus* activity was assessed. Enzymatic perturbation produced clear, strain-dependent effects (Table 1). For DD2, antimicrobial activity decreased after treatment with α-amylase and the proteases proteinase K and trypsin but was retained after lipase and pepsin treatment. This enzyme-response profile supports the involvement of both carbohydrate-associated and proteinaceous inhibitory component(s) in DD2 metabolites, consistent with the bacteriocin-like proteinaceous candidate detected in the downstream fractionation. DN1 showed a protease-sensitive pattern (reduced activity after proteinase K/trypsin/pepsin), suggesting that proteinaceous component(s) may also contribute to its inhibition of *C. rectus* growth. In contrast, DH1608 retained its inhibitory activity after all enzyme treatments, indicating that enzyme-resistant metabolite(s)—most plausibly organic acids and/or other small molecules—dominate its anti-*C. rectus* activity. DH5 displayed α-amylase sensitivity with otherwise limited effects of lipase/proteases, implying that carbohydrate-associated factor(s) may contribute alongside acid-mediated inhibition.

### Quantification of Organic Acids and Their Antimicrobial Activity

HPLC profiling of the kefir-derived LAB supernatants confirmed the presence of four target organic acids—citric acid (9.210 min), lactic acid (13.648 min), acetic acid (17.275 min), and propionic acid (20.814 min)—based on retention times of authentic standards (Supplementary Fig. S1). Quantification (Table 2) showed that lactic acid was the predominant organic acid in all kefir LAB supernatants, reaching 285.77 ± 9.68 mM (DD2), 359.83 ± 3.17 mM (DN1), 326.03 ± 6.11 mM (DH5), and 352.59 ± 12.02 mM (DH1608), all markedly higher than the levels in uninoculated MRS broth (41.30 ± 1.26 mM). Propionic acid was also consistently elevated in kefir LAB supernatants (26.15–28.11 mM) compared with uninoculated MRS (13.69 ± 4.70 mM). Acetic and citric acids showed strain-dependent variation, with acetic acid notably higher in DH5 (130.18 ± 0.59 mM) than that in MRS (108.88 ± 0.53 mM), while citric acid was generally reduced in kefir LAB supernatants relative to MRS (*e.g.*, DH1608: 5.43 ± 7.68 mM vs. MRS 23.75 ± 1.69 mM), which is consistent with citrate utilization during fermentation.

To verify that these organic acids contribute directly to the growth inhibition of *C. rectus*, lactic acid (300 mM) and propionic acid (25 mM) were tested individually at concentrations in the range quantified in the kefir LAB metabolites. Under the endpoint criterion used for the organic acid validation assay (no detectable growth at 24 h relative to the control), both lactic acid and propionic acid completely inhibited *C. rectus* growth. Collectively, these data indicate that the strong anti-*C. rectus* activity of the kefir-derived LAB supernatants is driven primarily by high lactic acid production, with propionic acid providing an additional inhibitory contribution within the measured concentration range.

### Quantification and Antimicrobial Activities of the Purified PP Fractions

As enzyme sensitivity experiments suggested that non-acid, proteinaceous components contribute to the anti-*C. rectus* activity of specific kefir isolates, purified PP fractions were prepared from each kefir-derived LAB supernatant and evaluated against *C. rectus*. DD2 produced the highest PP yield (12.58 ± 0.77 mg/L of MRS culture) and its PP fraction showed a clear and statistically significant inhibitory effect (48.16 ± 13.27%antimicrobial efficacy) compared with the buffer control (0%) (Table 3). PP fractions from DN1 and DH5 showed only minimal inhibition (approximately 14% efficacy), and DH1608 showed no detectable activity under the same assay conditions. PP yield was not proportional to antimicrobial efficacy across strains, suggesting that PP composition/structure rather than yield alone determines anti-*C. rectus* potency. Because PP fractions were tested at the maximum soluble concentration obtainable for each preparation, between-strain comparisons should be interpreted as semi-quantitative and reflect both concentration and intrinsic activity (see specific activity in Table 3).

To further characterize the DD2 PP fraction, which showed the strongest anti-*C. rectus* activity, the fraction was subjected to tricine–SDS-PAGE analysis. The analysis revealed a prominent band with an apparent molecular mass of approximately 28 kDa (Supplementary Fig. S2). Together with the protease sensitivity and strain-dependent loss of activity after heat treatment observed in the DD2 cell-free supernatant, this finding suggests the presence of proteinaceous antimicrobial component(s). However, the present results do not establish that the approximately 28-kDa band itself is responsible for the observed antimicrobial activity. Identification of the active component(s) will require further activity-guided purification and molecular characterization, such as LC–MS/MS analysis.

## Discussion

This study characterized kefir-derived LAB postbiotic metabolites that inhibit the growth of periodontal pathogen *C. rectus* and delineated the relative contribution of organic acids and a proteinaceous antimicrobial component to this inhibition. Across four kefir isolates (*L. kefiranofaciens* DD2 and DN1, *L. kefiri* DH5, and *L. mesenteroides* DH1608), the cell-free LAB supernatants suppressed *C. rectus* growth, with maximal inhibition observed when supernatants were produced at 30°C for 72 h. This production condition likely promotes the accumulation of inhibitory metabolites, particularly organic acids, while also enabling strain-specific production of non-acid inhibitory factors. Collectively, the data support a combined mechanism model in which acid-mediated inhibition is broadly important across strains, whereas DD2 additionally produces a protease-sensitive PP that has bacteriocin-like activity.

### Culture Condition (30°C, 72 h) as a Determinant of Anti-*C. rectus* Potency

A key practical finding is that the anti-*C. rectus* potency of the kefir-derived LAB postbiotic metabolites strongly depended on the culture conditions. The cell-free LAB supernatants produced at 30°C for 72 h consistently yielded the greatest growth inhibition of *C. rectus*, including the complete suppression of detectable growth by DD2 and DN1 in the microplate assay. A plausible explanation is that slower, extended culture at 30°C favors higher accumulation of organic acids and potentially other stress-induced antimicrobial metabolites. Kefir-associated LAB can exhibit strain-dependent growth and metabolite kinetics, and time-to-metabolite differences are well recognized among LAB cultures. For postbiotic development and scalable production, optimizing culture conditions to maximize metabolite output is essential because it directly links bioprocess parameters to antimicrobial functionality.

### Organic Acids Are the Dominant Baseline Inhibitor of *C. rectus*

HPLC profiling confirmed lactic acid as the dominant organic acid constituent of kefir-derived LAB postbiotics, with concentrations markedly elevated compared with uninoculated MRS. Propionic acid was also consistently elevated, while acetic and citric acids varied by strain. This pattern is consistent with the well-established role of LAB in organic acid production and the fact that acidic environments can constrain the growth of gram-negative bacteria, depending on acid identity and concentration. The antimicrobial activity of organic acids is commonly attributed to the ability of the undissociated acid to diffuse across membranes and dissociate intracellularly, thereby acidifying the cytoplasm, collapsing the proton motive force, and impairing nutrient transport and metabolism [34]. In addition, organic acid mixtures can produce additive or synergistic effects against susceptible organisms.

Critically, this study moved beyond correlation by directly demonstrating that lactic acid (300 mM) and propionic acid (25 mM) individually suppress *C. rectus* growth under an endpoint criterion, at concentrations within the quantified postbiotic range. This validation strengthens causal inference that organic acids are not merely biomarkers of fermentation but are functional drivers of anti-*C. rectus* activity. Propionic acid is often discussed for its inhibitory activity against fungi and certain bacteria, and its contribution here suggests that *C. rectus* may be particularly sensitive to short-chain organic acids beyond lactic acid. Although lactate likely provides the major inhibitory baseline due to its high concentration, propionate may provide an additional inhibitory layer that could be relevant when acid profiles shift across strains or processing conditions.

### Acid Stability and pH-Response Patterns Indicate Non-Acid Components in Selected Strains

The native pH of the kefir LAB supernatants (approximately 3.67–4.47 across the four isolates) reflects substantial acidification during culture, consistent with organic acid accumulation. When supernatants were adjusted to pH 2–9, their anti-*C. rectus* activities were largely retained at acidic pH (≤4), supporting acid-mediated inhibition as a robust mechanism. As expected, raising the pH diminished their inhibition of *C. rectus* because organic acids lose antimicrobial potency as the proportion of undissociated acid decreases [35, 36]. Notably, DH5 and DH1608 retained partial anti-*C. rectus* activity at basic pH (pH 8–9), suggesting that their supernatants contain inhibitory factor(s) that are not strictly dependent on low pH. This observation aligns with the broader view that LAB supernatants can contain multiple antimicrobial determinants—organic acids along with other metabolites such as hydrogen peroxide, ethanol, enzymes, or peptides—whose contribution can become more apparent when acid effects are reduced [37]. In a translational context, retention of partial activity of the kefir LAB supernatants after pH elevation is potentially relevant for oral applications where local pH can fluctuate due to saliva buffering and dietary inputs. However, because fully pH-matched control media and organic-acid-depleted preparations were not included, the contribution of non-acid antimicrobial factors should be interpreted cautiously. Therefore, the relative importance of acidic versus neutral metabolite-driven inhibition should be interpreted cautiously pending validation under physiologically relevant buffered conditions.

### Presence of a Heat-Labile Component in DD2 and DH5 Supernatants

Heat stability profiling revealed strain-dependent differences in postbiotic metabolite thermostability. DN1-derived metabolites retained high anti-*C. rectus* activity after heating at 100°C (>90%), and DH1608 retained measurable activity after heating at 100°C (residual efficacy >60%), which is consistent with inhibition dominated by thermostable small molecules such as organic acids. In contrast, DD2 and DH5 exhibited significant reductions after heating and autoclaving. Such a pattern suggests that, for these strains, inhibitory activity is not fully explained by organic acids alone and that an additional heat-labile component contributes meaningfully to overall potency.

From a postbiotic development viewpoint, thermal stability is a practical attribute: acid-driven activity is generally stable across many processing conditions, whereas proteinaceous antimicrobials can be partially or fully denatured depending on their structure. Importantly, the mixed stability profile observed here is not a drawback per se; rather, it helps partition mechanisms (acid vs. non-acid) and informs downstream steps (*e.g.*, choosing non-thermal processing if preserving peptide activity is desired).

### Enzyme Perturbation and PP Purification Converge on a Proteinaceous Inhibitor in DD2

Enzymatic sensitivity testing further characterized the biochemical nature of the non-acid inhibitory components in kefir-derived LAB postbiotics. DD2 activity decreased after α-amylase and protease (proteinase K and trypsin) treatments, indicating that both carbohydrate-associated and proteinaceous factor(s) may contribute to the anti-*C. rectus* activity of *L. kefiranofaciens* DD2. In contrast, DH1608 activity was largely unaffected by enzyme treatment, supporting a dominant role for enzyme-resistant metabolites (most plausibly organic acids and/or other small molecules). Such differential enzyme-response fingerprints are commonly used as a first-pass approach to classify antimicrobial determinants in cell-free supernatants and provide a rationale for targeted fractionation.

Guided by this logic, the PP fractions were purified from the kefir LAB supernatants. Among tested isolates, DD2 exhibited the highest PP yield and, notably, displayed the strongest antimicrobial activity (48.16 ± 13.27% efficacy) against *C. rectus*. The lack of proportionality between PP yield and antimicrobial efficacy across strains implies that PP bioactivity depends on their composition/structure rather than abundance alone, which is consistent with the diversity of bacteriocin-like proteins and the importance of sequence, post-translational features, or folding in determining their activity spectra. Bacteriocins and bacteriocin-like proteins are widely recognized as ribosomally synthesized antimicrobial peptides/proteins produced by bacteria that inhibit growth of other bacteria via mechanisms including membrane disruption, pore formation, and interference with essential processes [38–42]. Notably, antimicrobial efficacy values depend on both the intrinsic activity of each PP fraction and the final test concentration; therefore, potency ranking across strains should be interpreted cautiously despite normalization by specific activity.

### DD2 Polypeptide: A Bacteriocin-Like Antimicrobial Candidate Requiring Molecular Identification

Tricine–SDS-PAGE analysis of the DD2 PP fraction revealed a prominent band with an apparent molecular mass of approximately 28 kDa. However, the antimicrobial activity was demonstrated for the fraction as a whole, and the present results do not establish that the approximately 28-kDa band is the active component. Although the protease sensitivity and heat-labile characteristics suggest the involvement of proteinaceous antimicrobial component(s), their molecular identity and classification remain undetermined. Definitive identification will require further activity-guided purification followed by proteomic analysis, such as LC–MS/MS.

The next technical step should be activity-guided purification coupled with LC–MS/MS based proteomic identification of the active fraction, followed by targeted confirmation through approaches such as protease-sensitivity validation, heterologous expression (if feasible), or targeted genome mining for bacteriocin-biosynthesis loci. Mechanistically, high molecular weight, heat-labile bacteriocin-like molecules have been associated with pore formation and increased membrane permeability, but their modes of action are less comprehensively defined than those of smaller bacteriocins [41, 43]. Establishing identity and mechanism would substantially strengthen the translational positioning of the DD2 PP, including potential formulation strategies to preserve peptide activity.

### Implications for Kefir-Derived Postbiotics Targeting *C. rectus*

This work contributes to the emerging evidence that fermented food-derived postbiotics provide antimicrobial efficacy while circumventing the safety, stability, and regulatory complexities associated with live probiotic cultures [17, 18, 20]. The present findings indicate that kefir-derived LAB postbiotic metabolites inhibit *C. rectus* through combined organic acid-mediated and proteinaceous antimicrobial mechanisms. The first is the high production of lactic and propionic acids, which provides robust baseline inhibition, and the second is a *L. kefiranofaciens* DD2-derived, heat-labile proteinaceous factor (bacteriocin-like candidate) that offers complementary inhibitory activity. This dual mechanism is attractive for managing polymicrobial environments where reliance on a single antimicrobial determinant may be insufficient.

Translation to oral health applications requires careful consideration of context. Although acid-mediated inhibition is potent in vitro, persistent acidification in the oral cavity may compromise beneficial microbiota and enamel integrity. Future studies should evaluate whether effective anti-*C. rectus* activity is retained under buffered conditions mimicking oral pH, whether DD2 metabolites retain activity in saliva-containing matrices, and whether subinhibitory exposures modulate *C. rectus* physiology relevant to periodontal ecology. These questions are directly testable and would improve translational clarity of the study findings without changing the core mechanistic conclusions.

### Limitations and Future Directions

This study was primarily conducted in vitro and used cell-free LAB supernatants and fractions under controlled anaerobic conditions. While this approach is appropriate for mechanistic deconvolution, several avenues warrant further investigation. Future work should test activity of these cell-free LAB supernatants under saliva-mimicking buffering and protein conditions, and should quantify dose–response relationships (*e.g.*, minimum inhibitory concentration/minimum bactericidal concentration or time–kill kinetics) for both the organic acids and DD2 PP. In addition, the identity of the DD2 PP should be confirmed through proteomics/genomics, and the stability and activity of the organic acids and DD2 PP should be evaluated in relevant delivery formats. Finally, given the strain-specific behaviors observed herein, integrating metabolomics (broader than targeted organic acids) could reveal additional contributors and help explain why certain strains (*e.g.*, DH5/DH1608) retain partial activity at high pH while others do not. Some key limitations should also be acknowledged. The study was conducted in vitro only, and antimicrobial activity was not evaluated in saliva-like or buffered oral matrices. Although the antimicrobial activity of pH-adjusted supernatants was evaluated across pH 2–9, allowing partial assessment of activity under neutralized or alkalinized conditions, the present study did not include fully pH-matched control media or organic-acid-depleted preparations. Therefore, the relative contribution of acidification, specific organic acids, and non-acid antimicrobial factors could not be completely resolved. MIC, MBC, and time–kill kinetics were not determined. Although a representative tricine–SDS-PAGE image is provided, the DD2 proteinaceous antimicrobial candidate was not molecularly identified by LC–MS/MS or genomic analysis.

## Conclusion

Kefir-derived LAB produce cell-free metabolites that inhibit the periodontal pathogen *C. rectus* primarily through organic acid-mediated effects, with an additional protein-/polypeptide-associated contribution observed in the DD2 fraction. The DD2 protein-/polypeptide-enriched fraction showed antimicrobial activity, and tricine–SDS-PAGE revealed an approximately 28-kDa band within the fraction; however, the active component(s) were not molecularly identified. Further activity-guided purification and LC–MS/MS analysis are required for definitive identification and mechanistic characterization.

The demonstrated postbiotic functionality and stability advantages of kefir LAB metabolites position them as a promising candidate for further development in oral health applications. Critical next steps include molecular identification of DD2 polypeptide, assessment of activity retention under physiologically relevant buffered and saliva-containing conditions, and evaluation of dose-dependent effects relevant to periodontal ecology.

## Supplemental Materials

Supplementary data for this paper are available on-line only at http://jmb.or.kr.



## Figures and Tables

**Fig. 1:**
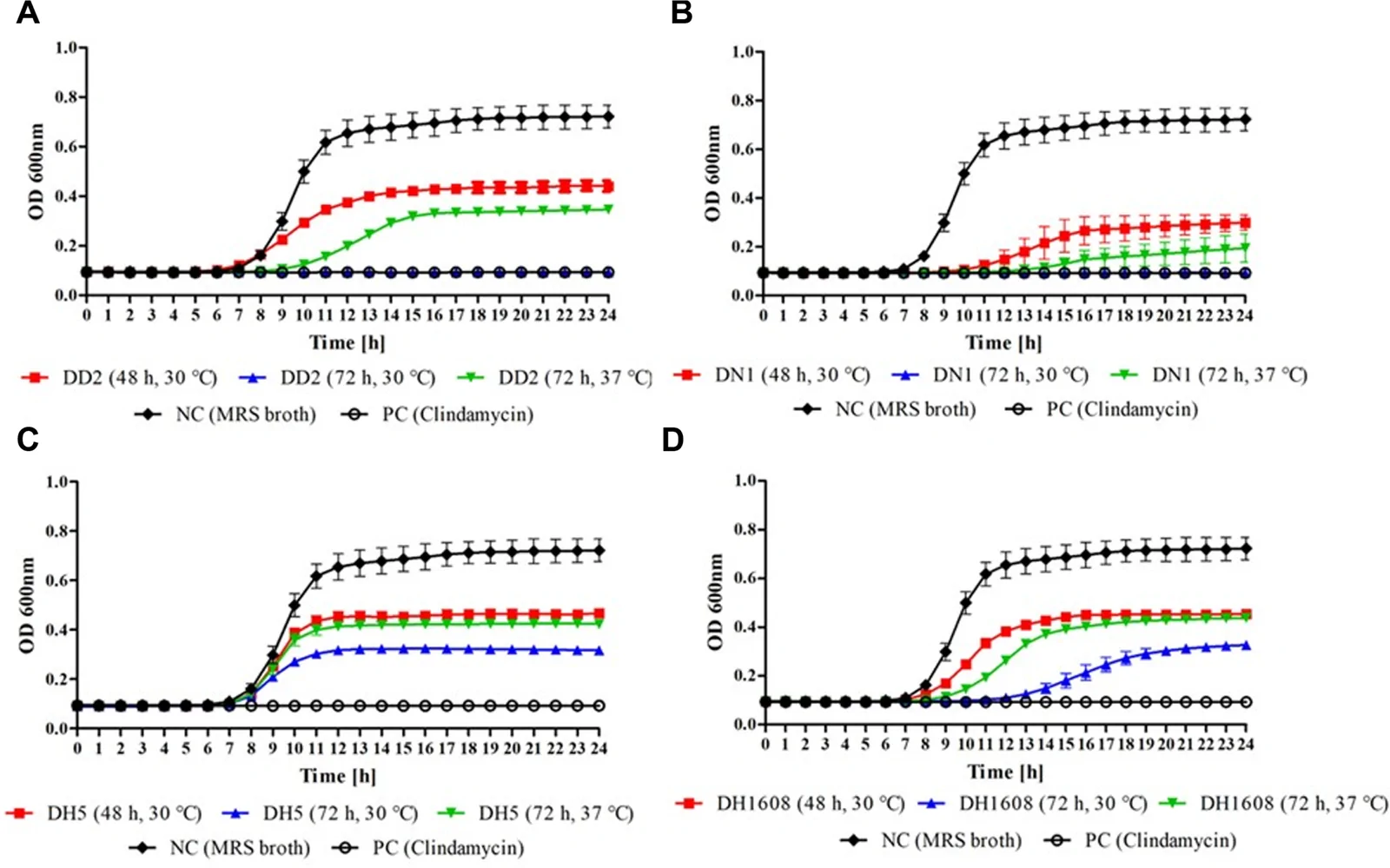
Growth kinetics of the periodontal pathogen *Campylobacter rectus* in nutrient broth supplemented with cell-free supernatants from kefir-derived lactic acid bacteria (LAB): (**A**) *Lactobacillus kefiranofaciens* DD2, (**B**) *L. kefiranofaciens* DN1, (**C**) *Lentilactobacillus kefiri* DH5, and (**D**) *Leuconostoc mesenteroides* DH1608. Cell-free supernatants were prepared under three incubation conditions (48 h at 30°C, 72 h at 30°C, and 72 h at 37°C) and evaluated using a 96-well growth-curve assay. Optical density at 600 nm (OD_600_) was measured hourly for 24 h. NC, negative control (de Man–Rogosa–Sharpe [MRS] broth); PC, positive control (clindamycin, 100 μg/mL). Values are presented as mean ± standard deviation (n = 3).

**Fig. 2:**
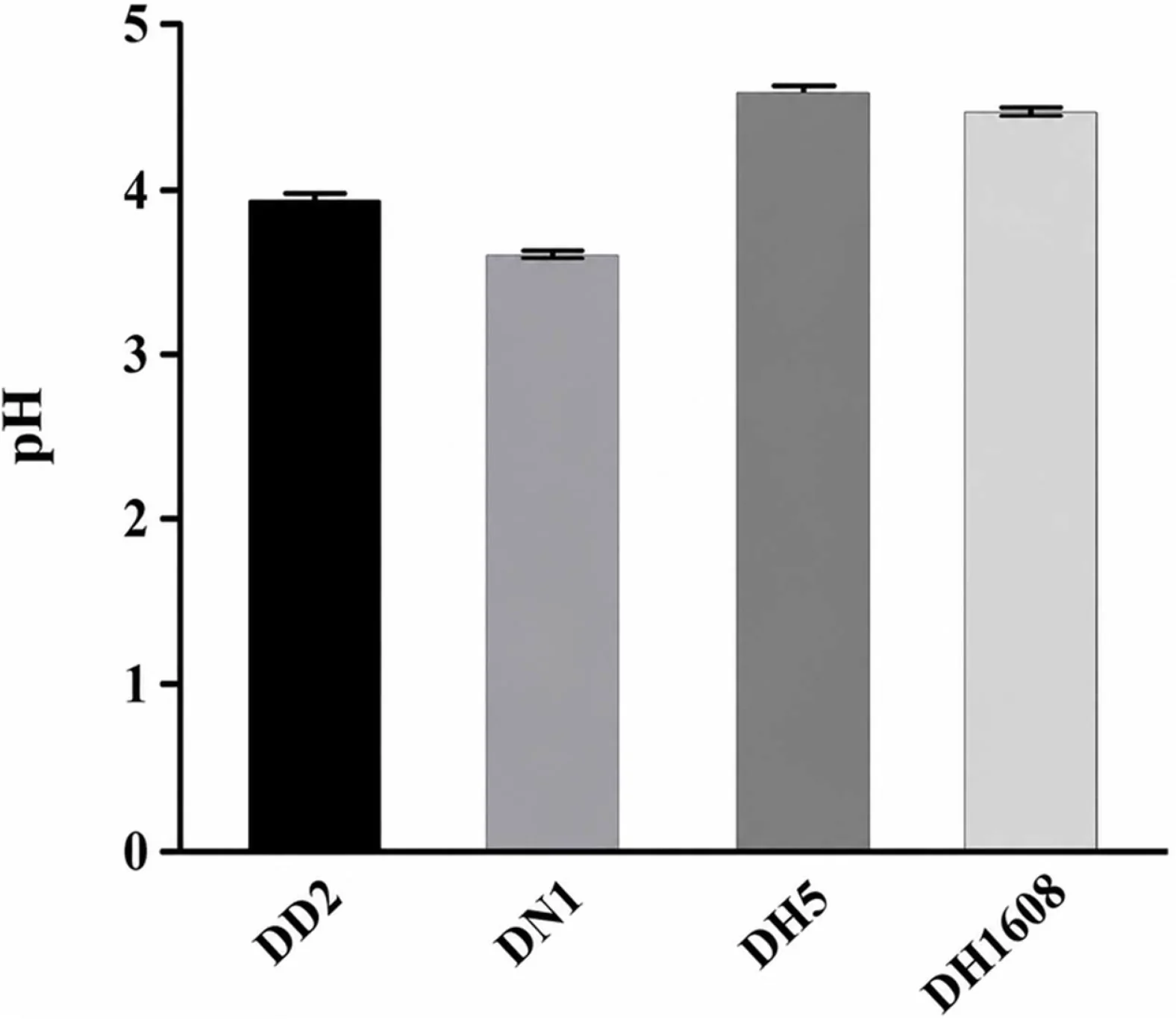
Native pH values of cell-free supernatants from kefir-derived lactic acid bacteria (LAB): *Lactobacillus kefiranofaciens* DD2 and DN1, *Lentilactobacillus kefiri* DH5, and *Leuconostoc mesenteroides* DH1608. Values are presented as mean ± standard deviation (n = 3).

**Fig. 3:**
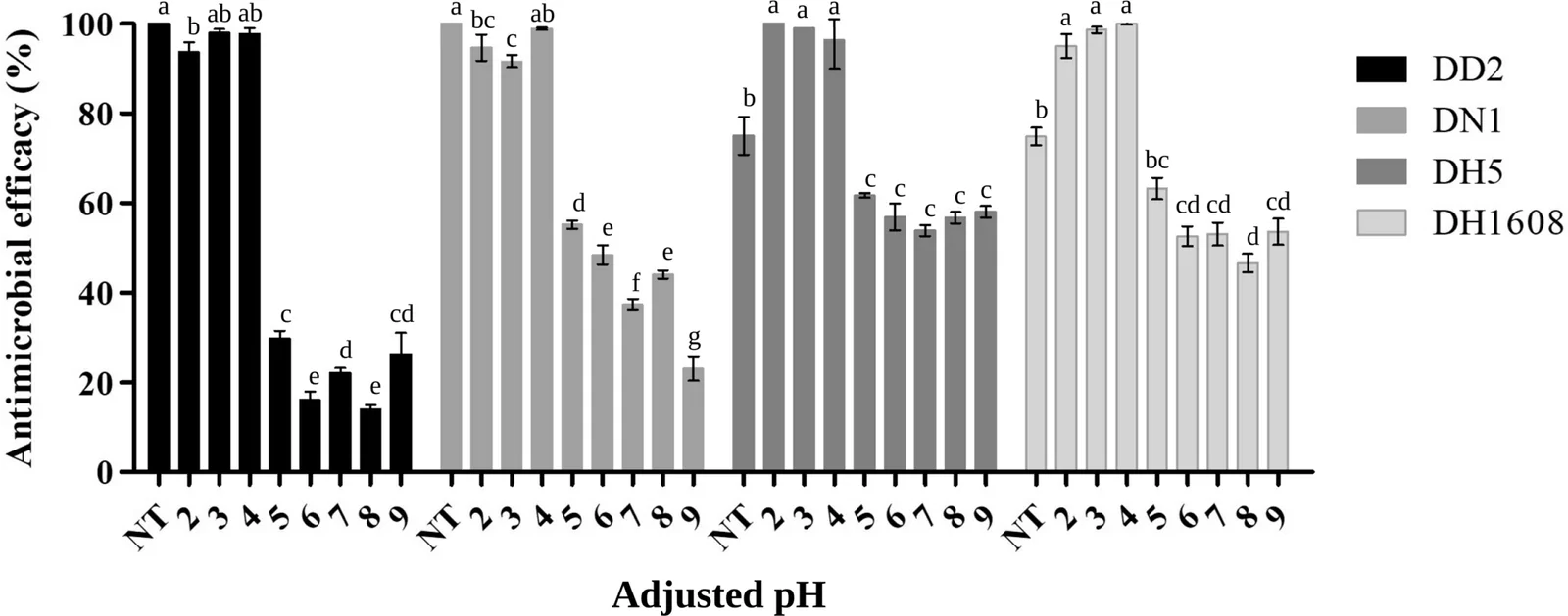
Antimicrobial efficacy of cell-free supernatants from kefir-derived lactic acid bacteria (LAB) against *Campylobacter rectus* following pH adjustment (pH 2–9). NT, untreated cell-free supernatant (without pH adjustment). For each strain, mean values with different letters differ significantly among pH treatments (*p* < 0.05). Values are presented as mean ± standard deviation (n = 3).

**Fig. 4:**
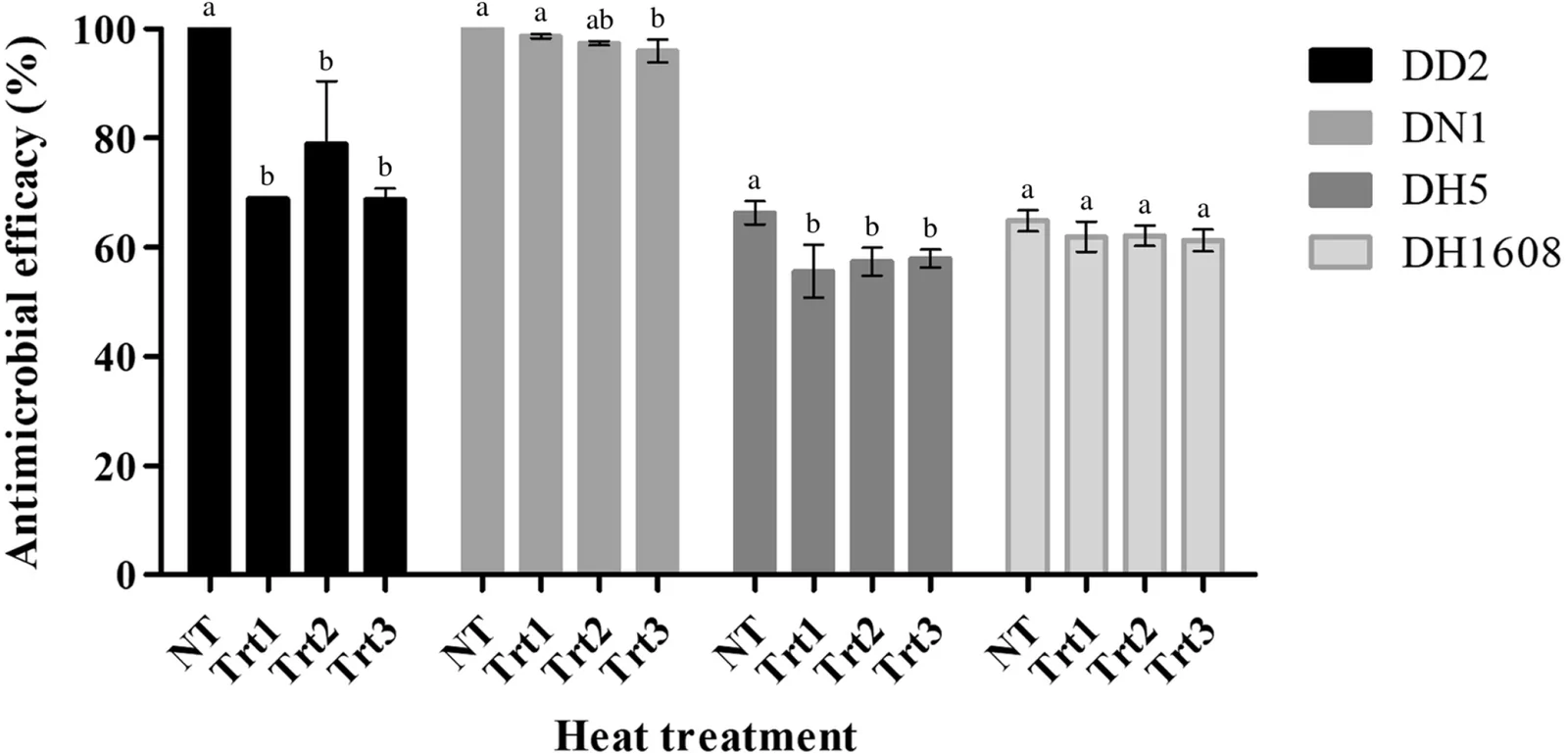
Antimicrobial efficacy of cell-free supernatants from kefir-derived lactic acid bacteria (LAB) against *Campylobacter rectus* following heat treatment. NT, untreated cell-free supernatant (no heat treatment); Trt1, 100°C for 10 min; Trt2, 100°C for 30 min; Trt3, 121°C for 15 min. For each strain, mean values with different letters differ significantly among heat treatments (*p* < 0.05). Values are presented as mean ± standard deviation (n = 3).

**Table 1:** Effect of enzyme treatment on the antimicrobial activity of kefir-derived lactic acid bacterial supernatants against *Campylobacter rectus*.

LAB strain	α-Amylase	Lipase	Proteinase K	Trypsin	Pepsin
*Lactobacillus kefiranofaciens* DD2	−	+	−	−	+
*L. kefiranofaciens* DN1	+	+	−	−	−
*Lentilactobacillus kefiri* DH5	−	+	+	+	+
*Leuconostoc mesenteroides* DH1608	+	+	+	+	+

−, *C. rectus* growth was observed in the growth-curve assay; +, no detectable *C. rectus* growth was observed.

**Table 2:** Organic acid concentrations in de Man–Rogosa–Sharpe (MRS) broth and cell-free supernatants of kefir-derived lactic acid bacteria.

Sample	Lactic acid (mM)	Propionic acid (mM)	Acetic acid (mM)	Citric acid (mM)
MRS broth	41.30 ± 1.26	13.69 ± 4.70	108.88 ± 0.53	23.75 ± 1.69
DD2	285.77 ± 9.68*	26.16 ± 5.77	105.48 ± 2.02	13.54 ± 3.39
DN1	359.83 ± 3.17*	26.15 ± 6.53	108.81 ± 1.72	19.72 ± 2.53
DH5	326.03 ± 6.11*	27.54 ± 8.04	130.18 ± 0.59*	12.97 ± 3.18
DH1608	352.59 ± 12.02*	28.11 ± 9.02	119.77 ± 6.78	5.43 ± 7.68*

^*^Significantly different from uninoculated MRS broth within the same organic acid column (*p* < 0.05).

DD2, *Lactobacillus kefiranofaciens* DD2; DN1, *Lactobacillus kefiranofaciens* DN1; DH5, *Lentilactobacillus kefiri* DH5; DH1608, *Leuconostoc mesenteroides* DH1608; LAB, lactic acid bacteria; MRS, de Man–Rogosa–Sharpe.

**Table 3:** Yield, test concentration, antimicrobial efficacy, and specific activity of purified polypeptide fractions from kefir-derived lactic acid bacterial supernatants against *Campylobacter rectus*.

LAB strain	Purified polypeptide yield (mg/L)	Final polypeptide concentration for antimicrobial test (mg/mL)	Antimicrobial efficacy vs. *C. rectus* (%)	Specific activity (% per mg/mL)
DD2	12.58 ± 0.77^a^	0.50 ± 0.18	48.16 ± 13.27^a^	96.32
DN1	9.20 ± 0.54^b^	0.74 ± 0.10	14.29 ± 1.84^b^	19.31
DH5	11.30 ± 1.32^a^	0.90 ± 0.09	14.29 ± 4.90^b^	15.88
DH1608	8.10 ± 0.50^b^	0.32 ± 0.01	0.00 ± 0.00^c^	0.00

Values represent mean ± standard deviation (n = 3). Within each column, values with different superscript letters differ significantly (one-way ANOVA with Tukey’s post hoc test, *p* < 0.05). Antimicrobial efficacy was calculated relative to the phosphate buffer control (0%). Polypeptide fractions were tested at the maximum soluble concentration after reconstitution; therefore, final test concentrations differed among strains. Specific activity was calculated as antimicrobial efficacy (%) divided by the final polypeptide concentration (mg/mL).

PP, polypeptide; DD2, *Lactobacillus kefiranofaciens* DD2; DN1, *Lactobacillus kefiranofaciens* DN1; DH5, *Lentilactobacillus kefiri* DH5; DH1608, *Leuconostoc mesenteroides* DH1608; LAB, lactic acid bacteria.
