# Supplementary material for: Kefir-Derived Postbiotics Inhibit the Periodontal Pathogen Campylobacter rectus through Combined Organic Acid and Proteinaceous Antimicrobial Mechanisms
Source: J Microbiol Biotechnol. 2026 Sep 21;36:e2607002. doi: 10.4014/jmb.2607.07002 (PMC13697206; doi:10.4014/jmb.2607.07002)
Supplement: Supplementary file 1 [file jmb-36-e2607002-supple.pdf]

J. Microbiol. Biotechnol. 2026. 36: e2607002
https://doi.org/10.4014/jmb.2607.07002

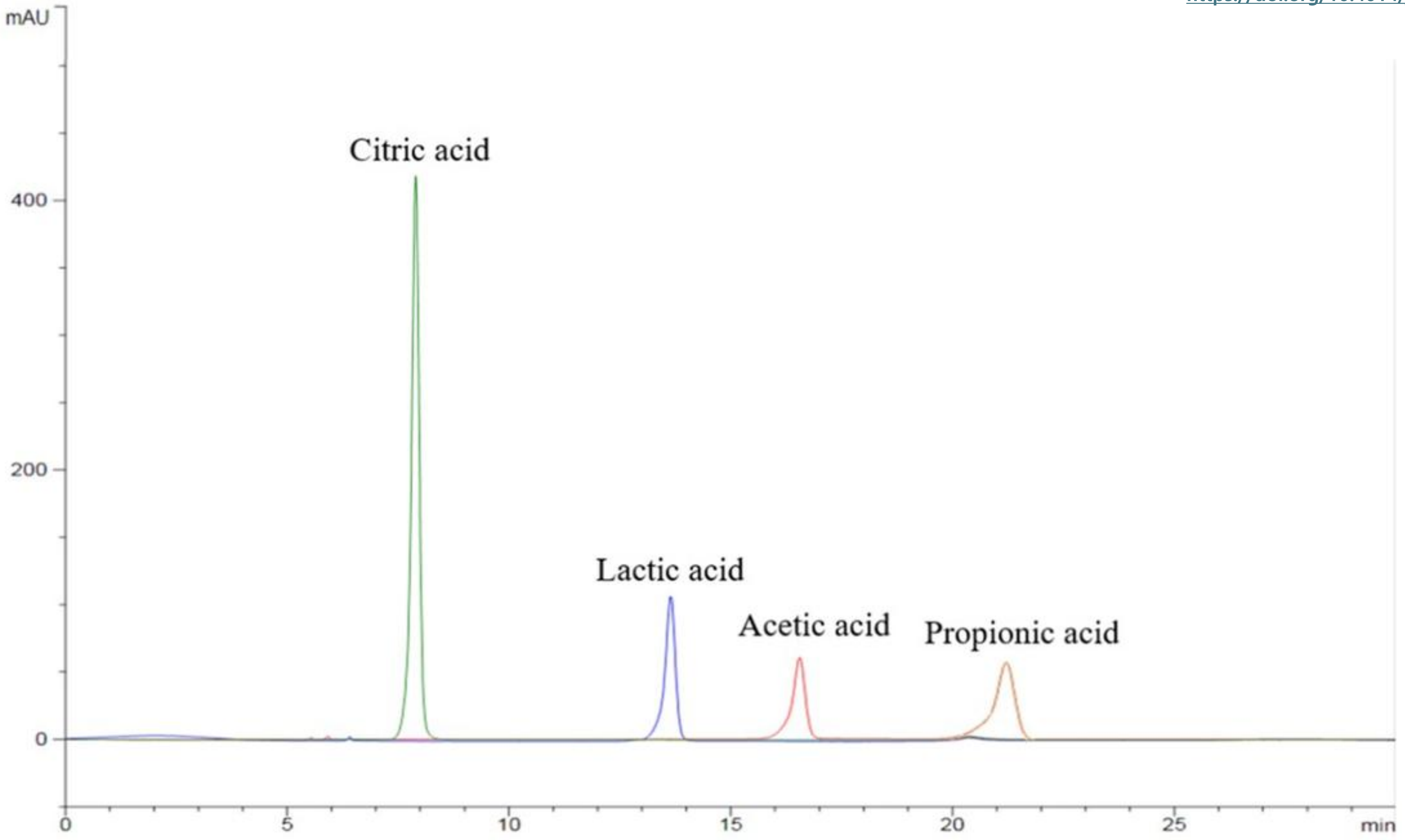

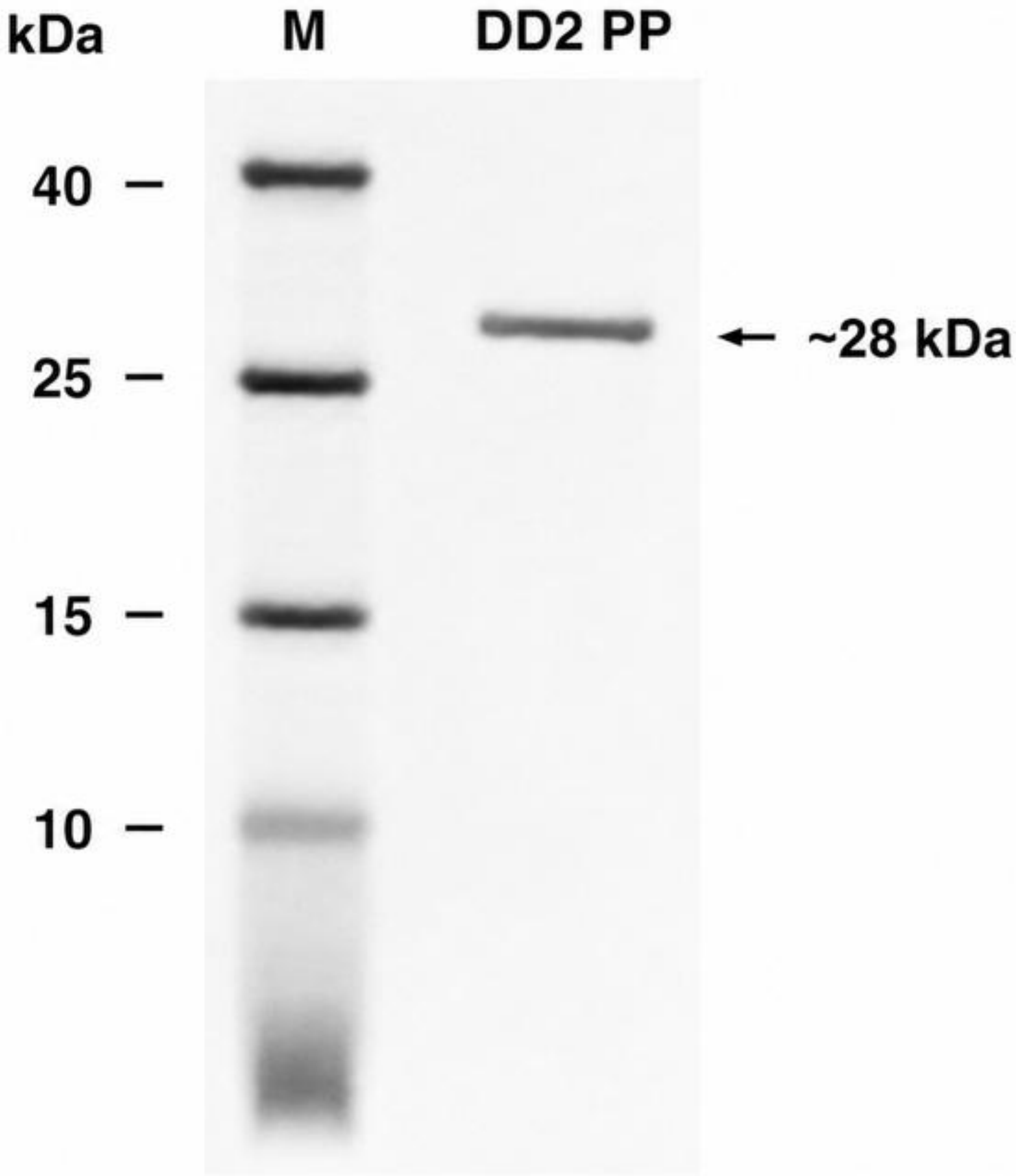
kDa
M
DD2 PP
40
25
15
10
~28 kDa
